# Dietary Patterns Associated with General Health of Breastfeeding Women 1–2 Months Postpartum: Data from the Japanese Human Milk Study Cohort

**DOI:** 10.1016/j.cdnut.2022.100004

**Published:** 2022-12-23

**Authors:** Satoshi Higurashi, Yuta Tsujimori, Keisuke Nojiri, Yasuhiro Toba, Kyoko Nomura, Hiroshi M. Ueno

**Affiliations:** 1 Research and Development Department, Bean Stalk Snow, Co., Ltd., Kawagoe, Japan; 2 Department of Environmental Health Science and Public Health, Akita University Graduate School of Medicine, Akita, Japan

**Keywords:** dietary pattern, maternal age, lactating women, undernourishment, Japan

## Abstract

**Background:**

The effects of dietary patterns on health outcome of lactating women remain unclear.

**Objectives:**

To describe the dietary patterns of lactating Japanese women and explore the association between dietary patterns and their general health.

**Methods:**

This study included 1096 lactating women from the Japanese Human Milk Study Cohort. The maternal diet during lactation 1–2 mo postpartum was determined using a FFQ. Dietary patterns were identified using a factor analysis based on the energy-adjusted intake of 42 food items. Trend associations between maternal and infant variables and quartiles of dietary pattern scores were tested, and logistic regression was performed to estimate the OR and 95% CI of maternal self-reporting anemia, constipation, rough skin, sensitivity to cold, and mastitis.

**Results:**

Four dietary patterns were identified in this study. The versatile vegetable diet, characterized by a high intake of vegetables, mushrooms, seaweeds, and tofu, was associated with maternal age, BMI prepregnancy and during the lactation periods, education, household income, and anemia. The plain Japanese diet contained a high intake of typical Japanese foods such as rice and miso soup and a low intake of bread and some confectioneries and was associated with maternal BMI during both periods. The salad vegetable diet, characterized by a high intake of raw vegetables and tomatoes with mayonnaise or dressing, was associated with parity and season in which data collection was conducted. The seafood diet, characterized by a high intake of fish, squid, octopus, shrimp, and shellfish, was associated with days postpartum and sensitivity to cold.

**Conclusions:**

Four dietary patterns were identified and were independently associated with socioeconomic factors. The versatile vegetables diet and seafood diet were associated with anemia and sensitivity to cold, respectively, among the participants. This trial was registered at the Japanese Clinical Trials Registry (https://center6.umin.ac.jp/cgi-open-bin/icdr_e/ctr_view.cgi?recptno=R000017649) as UMIN000015494.

## Introduction

Obesity is a growing health problem worldwide, with the prevalence of obesity nearly tripling since 1975 [1]. It is particularly severe in perinatal women, and obesity is not only associated with an increased risk of pregnancy complications, such as gestational hypertension and gestational diabetes, but has also been implicated in future metabolic abnormalities in the infant [2]. In contrast to these global trends, the increase in maternal underweight is a public health concern that is unique to Japan among other developed countries [3]. The number of underweight young women in Japan is increasing, reaching 25.2% of women aged 20–29 y [3], whereas only 2.2% of Canadian women aged 25–34 y are underweight [4]. Age-specific BMI has been steadily declining among Japanese adult women since the 1950s, and recently, age-specific BMI has ranked the lowest among selected countries [5]. Low preconception BMI is associated with an increased risk of fetal growth deficits, including lower birthweight. Low-birthweight infants accounted for 9.4% of all Japanese newborns in 2017, which was nearly twice than that in 1975 (5%) [3]. In addition, the average energy intake is below the estimated requirements for all generations of women [3]. Accordingly, Japanese women seem to manage their births and breastfeeding in a state of chronic undernourishment.

Breastfeeding mothers are at a high risk of undernourishment because of the additional nutritional requirements for maintaining breast milk supply. Human milk is the best source of nutrition for infants, and milk quality can be affected by maternal nutrition. Maternal intake of fatty acids and vitamins A, C, B-6, and B-12 influences the concentrations of these nutrients in breastmilk [6, 7]. Macronutrients are consistently present in mature milk and are less affected by maternal nutrition [8, 9]. Breastmilk supplies most nutrients to infants, thereby meeting their nutritional requirements, and breastmilk contains nutrients from the mother’s nutritional stores even under undernourished conditions [10].

In Japan, undernourished women may have an increased risk of various health issues during perinatal and breastfeeding periods. Anemia is a common health and nutritional problem worldwide, and women are particularly prone to anemia during menstruation, pregnancy, and childbirth. Sensitivity to cold is a clinical symptom in traditional oriental medicine, known as the common phenotype of “hiesho” in Japanese [11, 12]. Sensitivity to cold is prevalent in >50% of Japanese women [11] and is a possible risk factor for birth complications such as uterine inertia, prolonged labor, and postpartum hemorrhage [12]. The skin is among the tissues affected by the complex endocrinologic, immunologic, and metabolic changes during pregnancy [13], and rough skin is one of the most common postpartum concerns [14]. Mastitis is an inflammation of the breast tissue, such as the mammary gland, that occurs in lactating women. Mastitis is more prevalent during breastfeeding. Inflammation caused by mastitis not only causes distress to the mother but can also cause infants to avoid sucking from the affected breast [15]. Constipation is a major gastrointestinal symptom observed during the perinatal period, and breastfeeding women are at a high risk owing to the loss of fluids from milk production [16]. It is important to note that highly prevalent but not life-threatening health issues can worsen the quality of life of perinatal and lactating women [17].

All above-mentioned health issues are partly related to diets in healthy populations [[18], [19], [20], [21], [22]]. Constipation is inversely associated with the Japanese traditional diet, including a high intake of rice, miso soup, and soybean with limited intake of bread and confectionaries [19]. With some probiotic strains, no clinical signs of mastitis were reported after 14 d of intervention with dietary probiotics, with a decrease in staphylococcal count on day 30 after intervention [20]. Vitamins A, C, D, and E and trace minerals such as zinc, copper and selenium play an antioxidants role in maintaining skin health [21]. Some traditional herbs may be effective in the treatment of sensitivity to cold [[22], [23]]. These findings indicate an improvement in these health outcomes through dietary management.

WHO recommends that infants be exclusively breastfed for the first 6 mo of life. The Japanese national census shows increased breastfeeding rates over the past decade [24]. Undernourished women may frequently face health issues related to malnutrition because of the excess loss of nutrients from breast milk during lactation. Recently, a comprehensive approach to assess the overall diet, identifying dietary patterns was used to elucidate the relationship between dietary factors and health outcomes [25]. This approach considers complexity, including interactions between foods and nutrients, which cannot be examined in studies that focus on a single food and nutrient, and provides simple but insightful information [26, 27]. However, there are no reports regarding maternal anemia, constipation, rough skin, sensitivity to cold, and mastitis in Japanese women. Based on the above-described chronic undernourishment of Japanese women during the perinatal period, lactating women are at a higher risk of severe nutritional conditions, which may affect their health status. In consideration of the potential for dietary habits to change after childbirth, it is important to examine the dietary intake status during lactation, when nutritional requirements increase.

Therefore, in this study, we collected dietary data from Japanese lactating women 1–2 mo postpartum and determined the underlying dietary patterns with factor analysis. Furthermore, we explored the associations of these dietary patterns with sociodemographic and birth-related factors and maternal nutrient intake by correlation and trend tests. In addition, we investigated health outcomes such as maternal self-reporting anemia, constipation, rough skin, sensitivity to cold, and mastitis using logistic regression models.

## Methods

### Participants

This study was performed as a cross-sectional study in the early phase of the Japanese Human Milk Study Cohort [[28], [29], [30]]. Details of the recruitment of participants have been described previously [28]. In brief, lactating women and their infants were recruited after delivery across 73 medical institutions comprising 16 hospitals with the remaining institutions being obstetrics clinics across Japan between October 2014 and May 2019. Participants included in this study were healthy women with singleton infants of Japanese ethnicity and residency and who were willing to complete a dietary survey according to telephonically delivered and documented instructions.

### Dietary assessment

All study participants completed a questionnaire on food intake frequency at 1–2 mo postpartum, the time of their first dietary assessment for the Japanese Human Milk Study. A brief self-administered diet history questionnaire (BDHQ) was used to estimate the dietary intake of the lactating women. The BDHQ is a shortened version of the Diet History Questionnaire (DHQ) that can be easily used in the clinical setting and applied to pregnant women [[31], [32], [33]]. The BDHQ is a fixed-portion questionnaire that assesses habitual food intake and includes 58 food and beverage items during the preceding month. It includes questions on the frequency of consumption of foods and beverages commonly consumed in Japan and questions on cooking methods and general eating habits (Supplemental Table 1). The frequency of intake of each food items can be answered by selecting 1 of the 7 response options (e.g., for general food intake, the range was from “more than twice a day” to “rarely eat”). Standard portion sizes were derived from several recipe books for Japanese dishes [33]. Estimates of dietary intake were calculated using an ad hoc computer algorithm with weighting factors. Fifty-three of the 58 items of food and beverage were estimated based on reported intake, frequency of drinking, and fixed portion size, whereas the other 5 items, seasonings, fats and oils, and sugar intake, were estimated based on cooking methods and general eating habits [33]. Crude values of energy and nutrient intakes were estimated based on the calculated dietary intakes and the corresponding food composition lists in the Standard Tables of Food Composition of Japan [34]. The validity of the dietary intake data assessed with the BDHQ was confirmed through a study using 16-d semiweighted dietary records as the gold standard reference [31, 33]. The completed questionnaires were outsourced to the BDHQ’s provider, the DHQ Support Center (http://www.ebnjapan.org/), for the calculation of the dietary intake, energy, macronutrients, and micronutrients. We calculated the energy-adjusted intake of foods, beverages, and nutrients using the residual method to reduce the effects of systematic misreporting on dietary intake.

### Dietary pattern derivation

Dietary patterns were identified by a factor analysis using the maximum likelihood method. To determine the number of factors to retain, eigenvalues, a scree plot, a parallel analysis, and factor interpretability were considered. Several factors with eigenvalues of >1 existed; therefore, consideration was needed to determine the number of factors (Supplemental Table 1). First, a factor analysis was conducted using 48 foods and beverages of 58 items, excluding 5 items such as seasonings, which were calculated values, and 5 items of alcoholic beverages, which were consumed by <5% of the participants. According to the scree plot, the difference in eigenvalues among factors was constant, and no clear pattern could be found (Supplemental Figure 1A). A further factor analysis was conducted on 42 food items, excluding 6 items of nonalcoholic beverages from the 48 items. The results showed that the distribution of eigenvalues changed slightly; the scree plot decreased substantially after the fourth factor and remained similar thereafter (1.85 for the fourth factor, 1.59 for the fifth factor, and 1.54 for the sixth factor) (Supplemental Figure 1B), each of which was considered to represent a major dietary pattern of the Japanese; thus, we decided to retain the 4 factors. To simplify the structure, factor analysis was repeated while excluding dietary items with small values of factor loadings in any of the dietary patterns (the factor loading threshold was raised in steps from 0.2 to 0.3 in absolute value). Twenty-two dietary items were included in the final factor analysis, and parallel analysis supported the 4-factor structure (Supplemental Figure 1C). A comparison of the rotation methods of the factor analysis showed that the food items used were almost identical between the Varimax and Promax methods (data not shown). Therefore, the Promax method, which assumes a correlation among dietary patterns, was selected.

### Sociodemographic, anthropometric, and birth-related factors and maternal health status

A self-administered questionnaire including questions about the mother and infant prepared for the Japanese Human Milk Cohort was used to obtain information on factors such as sociodemographic characteristics (age, maternal education, household income, and family structure), anthropometrics (self-reported maternal current and prepregnancy BMI), smoking status, feeding methods, and birth-related environmental (delivery method, delivery experience, infant sex, and age) for the analysis (Supplemental Table 2) [28]. For recording health information, such as the weight of the infant and changes in the weight of the mother before and after pregnancy, we asked to refer to the Maternal and Child Health Handbook provided by the Japanese government [35]. In the questionnaire, participants were also asked about the frequency of anemia, constipation, rough skin, sensitivity to cold, and mastitis at 1–2 mo postpartum for common health issues during breastfeeding (e.g., “Please select the symptoms you are currently experiencing”). From the frequency responses obtained on a 5-point scale (1 = strongly disagree (never), 2 = disagree (rarely), 3 = neither agree nor disagree (occurs, but unaware in frequency), 4 = agree (sometimes), 5 = strongly agree (often or always)), a dichotomous scale was created by collapsing responses 1–3 from the original scale to 0 = asymptomatic and 4–5 to 1 = symptomatic. We asked the participants about the prevalence of anemia based on the reporting of subjective symptoms, such as fatigue, low appetite, and dizziness and a diagnosis by a doctor and/or blood test. The seasons during which the questionnaires were administered were also collected as a factor: spring = March to May, summer = June to August, autumn = September to November, and winter = December to February, according to the Japan Meteorological Agency classification.

### Statistical analysis

Data are presented as median (IQR) for continuous variables, in view of the skewed distribution (tested graphically and using the Shapiro–Wilk test) or sums (percentages) for categorical variables.

To determine the adequacy of the factor analysis, the Kaiser–Meyer–Olkin and Bartlett’s sphericity tests were used. Because the Kaiser–Meyer–Olkin value was 0.72 (above the acceptance criterion of 0.5) and *P* values for Bartlett’s sphericity test were <0.05, the model was judged to be valid. For each individual, the factor scores for dietary patterns were calculated as follows: the standardized intakes of the 22 food items used in the final factor analysis were weighted by factor loading and summed. Then, the sums were standardized. Participants were divided into quartiles based on the factor score of each dietary pattern, where the lowest (quartile 1) and highest (quartile 4) quartiles represented a low or high dietary pattern intake, respectively.

The variables were categorized according to the quartiles of dietary pattern scores. Trend associations between variables and quartiles of dietary pattern scores were tested using the Cochran–Armitage trend test for the nominal scale and the Jonckheere–Terpstra trend test for the ordinal scale and continuous variables. Correlations between dietary pattern scores and daily nutrient intake were evaluated using Spearman rank correlation coefficient. A logistic regression analysis was performed to estimate the OR and 95% CI of maternal health status during lactation, according to dietary patterns. Final multivariate adjusted ORs were calculated by adjusting for age, current BMI, education level, household income, delivery experience, and season when the questionnaires were collected, based on the statistical significance in the correlation and trend analyses. For the sensitivity analysis, multivariate models were developed using age, current BMI, education, and household income as covariates. The level of significance was set at a 2-sided *P* value of <0.05. All statistical analyses were performed using R (version 4.0.3; R Foundation for Statistical Computing) and EZR (Saitama Medical Center, Jichi Medical University), which are graphical user interfaces for R [36].

### Ethics

The study protocol was approved by the Internal Review Board of the Fukuda Clinic (approval number IRB20140621-03) and registered in the Japanese Clinical Trials Registry (UMIN000015494). All study procedures were performed in accordance with the principles of the 1975 Declaration of Helsinki, revised in 1983. All study participants provided written informed consent at the time of enrollment in the Japanese Human Milk Study, including for future use of biological data in published studies. Participants could withdraw from participation and continuation in the Japanese Human Milk Study at any time when health problems arose, and participants could consult with a health care professional. Participants who completed the questionnaire and provided breastmilk received a gift card for ∼27 USD (3000 Japanese yen). All data were anonymized at the creation of electrical data set based on the paper-based questionnaires and at the preparation of aliquots from the received frozen breastmilk provided by participants. The people in charge for managing personally identifiable data and anonymized data were different. The security of personally identifiable data was managed by the Director of Research and Development Department at Bean Stalk Snow, Co.

## Results

Of the 1122 participants in the entire cohort, 1096 participants responded to the questionnaire and dietary survey 1–2 mo postpartum and were included in this study (Supplemental Figure 2). The median age was 31 y, and the median gestational period was 39 weeks (Table 1). Most mothers were of normal weight (median prepregnancy BMI: 20.3 kg/m^2^; median current BMI: 21.5 kg/m^2^) and underwent vaginal delivery (88%). Single parity was observed in 34% of the women. Of the participants, 17.8% reported anemia, 45.9% constipation, 35.3% rough skin, 23.9% sensitive to cold, and 10.8% mastitis. Underweight was noted in 15.7% and 6.6% of the participants in prepregnancy and current periods. For infants, the median age was 58 d, and approximately half were boys (53.8%), with three-quarters (74.3%) being exclusively breastfed.

**TABLE 1:** Characteristics of lactating women

Characteristics	Total (*n* = 1096)
*Maternal characteristics*	
Age (y), median (IQR)	31 (28–34)
BMI, median (IQR)	
Prepregnancy	20.3 (19.1–22.1)
Current	21.5 (20.1–23.2)
Education, *n* (%)	
JHS/HS/Others	243 (22.2)
Some college/technical	460 (42)
4-y college/graduate degree	387 (35.3)
Household income (Jpy/y), *n* (%)	
<1,999,999	16 (1.5)
2,000,000–3,999,999	266 (24.3)
4,000,000–5,999,999	373 (34)
6,000,000–7,999,999	246 (22.4)
8,000,000–9,999,999	90 (8.2)
>10,000,000	61 (5.6)
Nuclear family, *n* (%)	960 (87.6)
Delivery experience, *n* (%)	
Once	378 (34.5)
Twice or more	713 (65.5)
Smoking habits, *n* (%)	
Never	759 (69.3)
Former	312 (28.5)
Current	21 (1.9)
Health conditions, *n* (%)	
Anemia	195 (17.8)
Constipation	503 (45.9)
Rough skin	387 (35.3)
Sensitivity to cold	262 (23.9)
Mastitis	118 (10.8)
Season when data were collected, *n* (%)	
Spring	280 (25.5)
Summer	252 (23)
Autumn	280 (25.5)
Winter	284 (25.9)
Birth and infant characteristics	
Cesarean delivery, *n* (%)	128 (11.7)
Gestational age (wk), median (IQR)	39 (38–40)
Infant’s sex (male), *n* (%)	590 (53.8)
Age (d), median (IQR)	58 (38–65.25)
Exclusive breastfeeding, *n* (%)	814 (74.3)

The values for categorical variables are shown as *n* (%) and those for continuous variables with a skewed distribution as the median (IQR). Variables are described as characteristics of the study settings that might affect the dietary intake or nutritional status of the participants [37]. HS, high school; JHS, junior high school; JPY, Japanese yen.

## Maternal dietary intake and patterns

We identified 4 components that were explained in 22 of the 42 food groups in the FFQ using the factor analysis with Promax rotation. Table 2 summarizes the factor loadings of the 4 patterns from food intake based on the FFQ, and Supplemental Table 3 indicates daily food intake by the quartile of dietary pattern score. The versatile vegetable pattern contained a high intake of vegetables, mushrooms, seaweed, and tofu. The plain Japanese pattern contained high intakes of typical Japanese foods such as rice and miso soup and low intakes of bread and some confectioneries. The salad vegetable pattern indicated a high intake of raw vegetables and tomatoes with mayonnaise or dressing. The seafood pattern was characterized by a high intake of fish, squid, octopus, shrimp, and shellfish.

**TABLE 2:** Factor loading matrix for major dietary patterns identified using factor analysis (*n* = 1096)

Food group		Versatile vegetable	Plain Japanese	Salad vegetable	Seafood
Carrots/pumpkin		0.85	−0.01	−0.11	−0.13
Japanese radish/turnip		0.70	0	−0.10	−0.06
Other root vegetables		0.68	−0.06	−0.03	−0.06
Mushrooms		0.61	0.01	−0.09	0.03
Cabbage/Chinese cabbage		0.58	0.01	0.15	−0.14
Green leaves vegetable		0.54	0.06	0.07	−0.01
Potatoes		0.36	−0.05	−0.01	0.13
Tofu/atsuage^1^		0.30	0.04	0.09	0.09
Seaweed		0.30	0.06	0.03	0.10
Rice		−0.30	0.98	−0.09	−0.34
Miso soup		0.07	0.39	−0.07	−0.04
Rice crackers/rice cake/okonomiyaki^2^		−0.06	−0.31	−0.03	−0.04
Ice cream		−0.12	−0.34	0.04	0.01
Bread		0.03	−0.41	0	−0.03
Western-type confectioneries		0.08	−0.43	−0.11	−0.14
Lettuces/cabbage (raw)		0.01	0.07	0.84	−0.07
Mayonnaise/dressing		−0.13	−0.04	0.67	−0.05
Tomatoes		0.10	−0.04	0.38	−0.01
Oily fish		−0.09	0	−0.04	0.65
Lean fish		0	0.05	−0.08	0.58
Dried fish/salted fish		−0.05	0.04	0	0.54
Squid/octopus/shrimps/shellfish		0.04	−0.05	0	0.31
Interfactor correlation	1	1			
	2	0.27	1		
	3	0.38	−0.06	1	
	4	0.45	0.22	0.20	1

1 Deep-fried tofu.

2 Meat/fish and vegetable pancake.

Correlations between dietary patterns and nutrient intake are presented in Table 3. The nutrients that had large correlation coefficients (>0.5, absolute value, *P* < 0.001) in each dietary pattern were as follows: The versatile vegetable pattern positively correlated with the intake of foods containing soluble dietary fiber (*r* = 0.782), insoluble dietary fiber (*r* = 0.827), total dietary fiber (*r* = 0.838), iron (*r* = 0.690), vitamin A (*r* = 0.59), vitamin B-1 (*r* = 0.716), vitamin B-6 (*r* = 0.727), vitamin C (*r* = 0.677), α-tocopherol (*r* = 0.585), α-carotene (*r* = 0.766), and β-carotene (*r* = 0.830). The plain Japanese dietary pattern inversely correlated with fat intake (*r* = −0.515). The salad vegetable pattern positively correlated with α-tocopherol intake (*r* = 0.512). Seafood consumption positively correlated with protein (*r* = 0.700), n–3 polyunsaturated fat (*r* = 0.716), sodium (*r* = 0.524), niacin (*r* = 0.695), vitamin B-6 (*r* = 0.585), vitamin B-12 (*r* = 0.873), and vitamin D (*r* = 0.812) intake.

**TABLE 3:** Spearman correlation coefficients between nutritional intake and dietary pattern scores (*n* = 1096)

Nutrient^1^	Daily intake	Versatile vegetable		Plain Japanese		Salad vegetable		Seafood	
		*r*	*P*	*r*	*P*	*r*	*P*	*r*	*P*
Protein (g)	67.18	0.450	<0.001	0.057	0.059	0.133	<0.001	0.700	<0.001
Fat (g)	58.22	0.144	<0.001	−0.515	<0.001	0.375	<0.001	0.284	<0.001
n–3 polyunsaturated fat (g)	2.50	0.275	<0.001	−0.069	0.022	0.335	<0.001	0.716	<0.001
n–6 polyunsaturated fat (g)	10.72	0.203	<0.001	−0.246	<0.001	0.451	<0.001	0.144	<0.001
Carbohydrate (g)	251.96	−0.208	<0.001	0.343	<0.001	−0.307	<0.001	−0.478	<0.001
Soluble dietary fiber (g)	3.02	0.782	<0.001	−0.079	0.009	0.234	<0.001	0.171	<0.001
Insoluble dietary fiber (g)	8.30	0.827	<0.001	0.075	0.014	0.249	<0.001	0.165	<0.001
Total dietary fiber (g)	11.66	0.838	<0.001	0.037	0.224	0.251	<0.001	0.183	<0.001
Calcium (mg)	517.85	0.467	<0.001	−0.145	<0.001	0.258	<0.001	0.318	<0.001
Iron (mg)	7.16	0.690	<0.001	0.093	0.002	0.238	<0.001	0.455	<0.001
Sodium (g, salt-equivalent)	9.74	0.231	<0.001	−0.007	0.825	0.129	<0.001	0.524	<0.001
Vitamin A^2^ (μg)	618.45	0.590	<0.001	−0.084	0.006	0.161	<0.001	0.260	<0.001
Vitamin B-1 (mg)	0.76	0.716	<0.001	−0.033	0.276	0.271	<0.001	0.403	<0.001
Vitamin B-2 (mg)	1.16	0.428	<0.001	−0.143	<0.001	0.192	<0.001	0.396	<0.001
Niacin (mg)	14.57	0.481	<0.001	0.022	0.467	0.132	<0.001	0.695	<0.001
Vitamin B-6 (mg)	1.16	0.727	<0.001	0.071	0.019	0.217	<0.001	0.585	<0.001
Vitamin B-12 (μg)	7.52	0.235	<0.001	0.041	0.175	0.052	0.084	0.873	<0.001
Vitamin C (mg)	94.59	0.677	<0.001	−0.065	0.032	0.299	<0.001	0.241	<0.001
Vitamin D (μg)	10.92	0.263	<0.001	0.070	0.020	0.044	0.146	0.812	<0.001
α-tocopherol (mg)	7.34	0.585	<0.001	−0.301	<0.001	0.472	<0.001	0.391	<0.001
β-tocopherol (mg)	0.39	0.154	<0.001	−0.400	<0.001	0.390	<0.001	0.046	0.127
γ-tocopherol (mg)	13.22	0.212	<0.001	−0.229	<0.001	0.512	<0.001	0.110	<0.001
δ-tocopherol (mg)	3.28	0.310	<0.001	−0.065	0.031	0.361	<0.001	0.147	<0.001
α-carotene (μg)	410.66	0.766	<0.001	0.021	0.494	0.074	0.014	0.069	0.022
β-carotene (μg)	2992.61	0.830	<0.001	0.036	0.238	0.243	<0.001	0.130	<0.001
β-cryptoxanthin (μg)	197.99	0.179	<0.001	−0.125	<0.001	0.038	0.212	0.194	<0.001

1 Nutrient intake was adjusted for energy intake using the residual method. For daily nutrient intake by the quartile of dietary pattern score, see Supplemental Table 4.

2 Retinol equivalent.

### Associations between dietary patterns and demographic characteristics

Table 4 presents the lowest (quartile 1) and highest (quartile 4) quartiles of each dietary pattern in the dyads. The versatile vegetable pattern group tended to be older (*P*-trend = 0.021) and reported lower prepregnancy and current BMI (*P*-trend = 0.010 and 0.034, respectively), higher education level (*P*-trend < 0.001), and higher household income (*P*-trend = 0.002). The plain Japanese pattern group reported higher prepregnancy and current BMI (*P*-trend = 0.034 and 0.030, respectively). Participants with higher scores for the salad vegetable pattern showed a higher proportion of single parity (*P*-trend < 0.001). Moreover, participants with higher scores for the seafood pattern reported a lower infant age (*P*-trend = 0.014). The salad vegetable pattern was found to be affected by season, with higher scores in summer and lower scores in winter (*P*-trend < 0.001).

**TABLE 4:** Characteristics of lactating women for the lowest (Q1) and highest (Q4) quartiles of 4 dietary patterns^1^

Characteristics	Versatile vegetable			Plain Japanese			Salad vegetable			Seafood		
	Q1	Q4	*P*	Q1	Q4	*P*	Q1	Q4	*P*	Q1	Q4	*P*
*n*	274	274		274	274		274	274		274	274	
Lactating women												
Dietary score, median	−0.96	1.07	<0.001	−1.00	1.12	<0.001	−0.98	1.03	<0.001	−0.95	1.07	<0.001
Age (y), median	31	32	0.021	32	32	0.932	31	31	0.852	31	31	0.150
BMI, median												
Prepregnancy	20.6	20.0	0.010	20.1	20.6	0.034	20.3	20.3	0.694	20.6	20.2	0.165
Current	21.8	21.3	0.034	21.2	21.9	0.030	21.5	21.5	0.960	21.5	21.4	0.429
Education (%)			<0.001			0.320			0.555			0.229
JHS/HS/others	31	18		21	25		26	22		24	21	
Colleges/technical	43	40		41	41		41	41		44	43	
4-y College/graduate	26	42		38	34		34	36		31	36	
Household income (Jpy/y), %			0.002			0.545			0.060			0.274
<1,999,999	1.5	1.9		2.7	0.8		1.9	1.9		1.1	1.9	
2,000,000–3,999,999	34	23		22	25		28	20		31	23	
4,000,000–5,999,999	32	34		39	34		36	32		32	39	
6,000,000–7,999,999	23	22		24	25		23	29		24	23	
8,000,000–9,999,999	6.5	9.6		6.5	6.8		7.2	11		9.2	6.4	
>10,000,000	3.0	8.4		6.1	7.6		4.6	5.4		3.4	6.8	
Nuclear family (%)	10	14	0.779	12	13	0.592	12	12	0.902	11	12	0.383
Delivery experience (%)			0.725			0.488			<0.001			0.187
One time	33	36		36	34		28	42		32	37	
Twice or more	67	64		64	66		72	58		68	63	
Smoking habits (%)			0.133			0.862			0.424			0.605
Never	67	73		72	73		71	68		69	74	
Former	30	25		28	25		26	30		29	24	
Current	2.6	2.2		0.7	2.6		2.6	2.6		1.8	1.8	
General health (%)												
Anemia	22	16	0.015	20	17	0.284	18	15	0.343	22	19	0.320
Constipation	44	45	0.757	46	44	0.288	49	40	0.039	49	49	0.838
Rough skin	36	36	0.977	32	38	0.384	38	34	0.253	39	32	0.157
Sensitivity to cold	26	24	0.731	27	24	0.614	31	22	0.005	32	22	0.004
Mastitis	12	11	0.907	11	13	0.305	14	10	0.226	10	12	0.426
Season (data were collected), %			0.452			0.158			<0.001			0.166
Spring	31	24		27	27		23	25		30	23	
Summer	25	20		28	19		13	35		22	25	
Autumn	24	26		23	26		28	24		22	26	
Winter	20	31		21	28		36	15		26	26	
Birth and infant characteristics	7	6		3	7		5	5		7	6	
Cesarean delivery (%)	12	11	0.992	14	9.2	0.052	12	9.2	0.322	13	12	0.851
Gestational age (wk), median	39	39	0.289	39	39	0.803	39	39	0.439	39	39	0.711
Infant sex (male), %	58	51	0.175	57	56	0.957	58	54	0.416	56	55	0.626
Age (d), median	56	53	0.118	53	59	0.473	60	56	0.117	58	50	0.014
Exclusive breastfeeding (%)	72	75	0.264	74	71	0.172	74	75	0.889	71	78	0.066

1 The values for categorical variables are shown as (%) and those for continuous variables with a skewed distribution as median. Trend associations between variables and quartiles of dietary pattern scores were tested using the Cochran–Armitage trend test for the nominal scale and the Jonckheere–Terpstra trend test for the ordinal scale and continuous variables. For complete data, see Supplemental Table 5. HS, high school; JHS, junior high school; JPY, Japanese yen.

### Associations of dietary patterns with maternal anemia, sensitivity to cold, constipation, and rough skin

Associations between dietary patterns and maternal health status were examined using a multivariate logistic regression analysis. Table 5 shows the adjusted ORs (95% CIs) for maternal anemia, sensitivity to cold, constipation, and rough skin according to dietary patterns. Maternal anemia was inversely associated with the versatile vegetable pattern (adjusted OR: 0.829; 95% CI: 0.696, 0.986; *P* = 0.035). Sensitivity to cold was inversely associated with the seafood pattern (adjusted OR: 0.818; 95% CI: 0.698, 0.958; *P* = 0.013). Maternal constipation and rough skin were not associated with any dietary patterns, whereas some associations were observed only in the crude univariate models.

**TABLE 5:** OR from univariate and multivariate logistic regression analyses evaluating associations between dietary patterns and maternal general health^1^

Variables	Versatile vegetable		Plain Japanese		Salad vegetable		Seafood	
	OR (95% CI)	*P*	OR (95% CI)	*P*	OR (95% CI)	*P*	OR (95% CI)	*P*
Anemia								
Unadjusted	0.835 (0.706, 0.988)	0.036	0.880 (0.752, 1.03)	0.113	0.905 (0.770, 1.06)	0.228	0.965 (0.824, 1.13)	0.660
Model 1	0.829 (0.698, 0.984)	0.033	0.883 (0.751, 1.04)	0.130	0.901 (0.764, 1.06)	0.218	0.940 (0.799, 1.11)	0.456
Model 2	0.829 (0.696, 0.986)	0.035	0.884 (0.751, 1.04)	0.136	0.875 (0.740, 1.04)	0.123	0.915 (0.774, 1.08)	0.293
Constipation								
Unadjusted	0.903 (0.800, 1.02)	0.101	0.957 (0.848, 1.08)	0.469	0.882 (0.780, 0.996)	0.043	1.03 (0.915, 1.16)	0.609
Model 1	0.910 (0.803, 1.03)	0.137	0.941 (0.832, 1.06)	0.332	0.888 (0.784, 1.01)	0.062	1.04 (0.916, 1.17)	0.570
Model 2	0.911 (0.804, 1.03)	0.146	0.940 (0.831, 1.06)	0.328	0.891 (0.785, 1.01)	0.073	1.05 (0.925, 1.19)	0.458
Rough skin								
Unadjusted	1.01 (0.889, 1.14)	0.912	1.05 (0.930, 1.19)	0.416	0.955 (0.842, 1.08)	0.474	0.870 (0.763, 0.991)	0.036
Model 1	1.00 (0.881, 1.14)	0.972	1.06 (0.930, 1.2)	0.397	0.953 (0.837, 1.08)	0.461	0.871 (0.762, 0.995)	0.043
Model 2	1.02 (0.893, 1.16)	0.805	1.07 (0.937, 1.21)	0.329	0.924 (0.810, 1.05)	0.239	0.880 (0.768, 1.01)	0.063
Sensitivity to cold								
Unadjusted	0.991 (0.859, 1.14)	0.902	0.98 (0.852, 1.13)	0.779	0.854 (0.737, 0.99)	0.037	0.810 (0.695, 0.944)	0.007
Model 1	0.983 (0.850, 1.14)	0.822	0.979 (0.848, 1.13)	0.771	0.851 (0.731, 0.991)	0.038	0.824 (0.705, 0.962)	0.015
Model 2	0.969 (0.835, 1.12)	0.675	0.964 (0.833, 1.12)	0.621	0.871 (0.746, 1.02)	0.081	0.818 (0.698, 0.958)	0.013
Mastitis								
Unadjusted	0.985 (0.812, 1.20)	0.880	1.14 (0.948, 1.38)	0.160	0.843 (0.684, 1.04)	0.107	1.05 (0.872, 1.27)	0.600
Model 1	0.973 (0.796, 1.19)	0.787	1.17 (0.963, 1.41)	0.115	0.844 (0.680, 1.05)	0.124	1.03 (0.850, 1.26)	0.737
Model 2	0.984 (0.805, 1.20)	0.873	1.17 (0.970, 1.42)	0.100	0.817 (0.656, 1.02)	0.071	1.04 (0.856, 1.27)	0.677

1 Model 1: adjusted for age, current BMI, education level, and household income. Model 2: adjusted for age, current BMI, education level, household income, delivery experience, and season.

## Discussion

In this study, 4 dietary patterns were identified among lactating women in Japan. We examined the dietary patterns and their association with the maternal health outcomes including anemia, constipation, rough skin, sensitivity to cold, and mastitis. Anemia, constipation, sensitivity to cold, rough skin, mastitis, and low maternal BMI were common among study participants. Maternal anemia was inversely associated with the versatile vegetable pattern, and sensitivity to cold was inversely associated with the seafood pattern in the multivariate models. There is a lack of information on the nutritional status of lactating women, and the results of this study provide information for the establishment of appropriate nutritional guidance, particularly in Japan, where improvement of undernutrition is a social challenge in some populations.

The reported prevalence of anemia (defined as hemoglobin concentrations <12.0 g/dL) among Japanese women aged 20–49 y was 17.1% [38], which is comparable with that found in the present study (17.8%). Anemia is caused by a decrease in iron stores in the body. Although the versatile vegetable and seafood patterns were associated with iron intake, only the versatile vegetable pattern was inversely associated with the prevalence of anemia. The versatile vegetable pattern contains nonheme iron and ascorbic acid from nonstarchy vegetables, seaweed, and soybean products, typically found in washoku, a traditional Japanese diet [39]. The improved bioavailability of nonheme iron with ascorbic acid reduces the risk of anemia, despite the lower absorption of nonheme iron than of heme iron [40]. A well-balanced diet is essential for maintaining stored iron and preventing anemia.

The proportion of participants with symptoms of sensitivity to cold was low (23.9%) in this study compared with the reported prevalence of >50% [41]. Sensitivity to cold may indirectly affect uterine inertia and prolong labor, which are known risk factors for postpartum hemorrhage and associated birth problems [12]. The participants in this study are likely to be healthier than the general population because of the recruitment of almost all participants through local clinics [28]. This may have resulted in a lower prevalence of sensitivity to cold than that previously reported. Sensitivity to cold may also be caused by circulatory disturbances related to peripheral arterial vasoconstriction and autonomic dysfunction [41]. Seafood is abundant in polyunsaturated fatty acids and antioxidant vitamins such as γ-tocopherols. A high intake of antioxidant nutrients was attributed to improved blood circulation and autonomic nerve balance. Alternatively, patients with iron-deficiency anemia may complain of cold intolerance [42], and the clinical symptoms of B-12 deficiency result primarily from pernicious anemia [43]. Patients with B-12 deficiency reported improvement in symptoms of cold intolerance after 6 mo of hydroxocobalamin therapy [44]. There is a high prevalence of vitamin B-12 insufficiency among populations consuming low amounts of animal-source foods. Vitamin B-12 and iron were the first and second influenced factors, respectively, loaded in the seafood diet among the 4 dietary patterns identified in this study. The nutritional status of patients with iron and vitamin B-12 deficiency may play a role in the finding of sensitivity to cold associated with the seafood pattern.

In addition, some possible associations were observed in the crude univariate models. Maternal constipation was inversely associated with the salad vegetable pattern (unadjusted OR: 0.882; 95% CI: 0.780, 0.996; *P* = 0.043; adjusted OR: 0.891; 95% CI: 0.785, 1.01; *P* = 0.073). Rough skin was inversely associated with seafood consumption (unadjusted OR: 0.870; 95% CI: 0.763, 0.991; *P* = 0.036; adjusted OR: 0.880; 95% CI: 0.768, 1.010; *P* = 0.063). In addition, mastitis was weakly inversely associated with the salad vegetable pattern (adjusted OR: 0.817; 95% CI: 0.656, 1.020; *P* = 0.071) and weakly positively associated with the plain Japanese pattern (adjusted OR: 1.170; 95% CI: 0.970, 1.420; *P* = 0.100). These diets may partly involve the rough skin and mastitis in the lactating women through dietary exposure.

Although limited information is available about the dietary patterns of lactating Japanese women, the versatile vegetable and plain Japanese patterns identified in this study were similar to the healthy and Japanese patterns identified in previous studies [45, 46]. In these studies, the foods related to salad vegetable and seafood, which formed independent patterns in this study, were included in the “healthy” pattern. In addition, the “western” pattern, characterized by meat and egg consumption, was not found in this study. A Western pattern was identified as the third most popular pattern in a cross-sectional study involving female university students in Japan, whereas the extent of diet westernization appeared smaller to the Polish counterparts in the same study [47]. Another westernized pattern, characterized by frequent intake of bread but infrequent intake of rice for breakfast, is inversely related to A1C concentrations in Japanese men and women [48]. Frequent consumption of rice was identified in the plain Japanese pattern in this study, partly reflecting the preference of grains as staple foods. Sex-based differences in dietary behaviors were studied in a large cross-sectional study involving 84988 Japanese adolescents from the seventh to 12th grades. Among girls, there was an inverse association with skipping breakfast, snacking, eating out, skipping meals, and eating alone at dinner, although a stronger association with subjectively poor diet quality was found among girls than in boys [49]. Accordingly, a western diet was potentially identified, but sensitive perception on diet quality would affect our results with no apparent western patterns. Taken together, the 4 dietary patterns identified in this study reflect stereotypic diets for Japanese women during lactation; however, they may also be influenced by other factors, such as the environment. The salad vegetable pattern was affected by seasons; this pattern showed an opposite trend, as strong in summer and weak in winter. The results of a meta-analysis of 6 studies examining the association between different food groups and season showed that season is a major determinant of fruit, vegetable, egg, meat, cereals, and alcoholic beverages intake, with vegetable intake being particularly high in summer [50]. In addition, a Japanese study found that vegetable and dairy intake is higher in summer [51]. These findings support the seasonal changes in salad vegetable patterns observed in this study.

The strength of this study is that the dietary survey was conducted within a narrow period of 1–2 mo postpartum, minimizing the effect of longitudinal changes in nursing behavior. A previous study found that dietary patterns did not change during pregnancy [52] but did not elucidate any further changes during the breastfeeding period. In this study, the salad vegetable pattern was influenced by delivery experience (multiparity), suggesting that dietary patterns are different according to the presence of siblings or with the growth of their children. In addition, the infants tended to be younger in the group with higher scores on the seafood pattern, suggesting that mothers with infants consciously consume seafood. Our previous cross-sectional study found that milk docosahexaenoic acid concentrations are inversely associated with infant age and maternal seafood consumption [29]. Further follow-up can expand the longitudinal changes through continuous dietary surveys in this cohort.

This study has some limitations. First, because of the nature of the cross-sectional study, we could not determine the cause–effect relationships in this study. Second, maternal health status was subjectively assessed using a self-administered questionnaire developed for the purpose of this study. Therefore, further studies are required to validate the questionnaire in clinical practice. The questionnaire did not have clear diagnostic criteria for health-related items; thus, anemia, constipation, rough skin, sensitivity to cold, and mastitis were selectively incorporated in this study owing to the nature of the cohort consisting of healthy lactating women [28]. This also meant that the results could not be assumed to apply to the general population of lactating Japanese women. Third, dietary intake was assessed using the FFQ. Although the validity of the FFQ (BDHQ) has been confirmed, the estimated intake of nutrients and food cannot be completely reflected by dietary habits. Lastly, although we adjusted for several potential independent covariates, unmeasured confounders, such as genome and gut microbiome in the study participants, related to maternal health status may have remained in this study.

In conclusion, we identified 4 main dietary patterns in lactating women 1–2 mo postpartum in Japan: versatile vegetable, plain Japanese, salad vegetable, and seafood patterns. These patterns were associated with maternal age, BMI, education, household income, delivery experience, season, and infant age. The versatile vegetable and seafood diet were associated with anemia and sensitivity to cold, respectively, among the participants.

## Author disclosures

SH, HMU, YTsujimori, KNojiri, and YToba were employees of Bean Stalk Snow. Bean Stalk Snow manufactures and sells dietary supplements for perinatal women. Megmilk Snow Brand manufactures and sells dairy foods. KNomura reports no conflicts of interest. These organizations did not conduct any violations, interferences, or substantial influences on the scientific observations and conclusions in this study.

## Data Availability

The data described in the manuscript, code book, and analytic code will be made available on request pending approval.

## Appendix A Supplementary data

Multimedia components 1 Multimedia components 1

Multimedia components 2 Multimedia components 2
