# Supplementary material for: Dietary Patterns Associated with General Health of Breastfeeding Women 1–2 Months Postpartum: Data from the Japanese Human Milk Study Cohort
Source: Curr Dev Nutr. 2022 Dec 23;7(1):100004. doi: 10.1016/j.cdnut.2022.100004 (PMC10064480; doi:10.1016/j.cdnut.2022.100004)
Supplement: Multimedia components 1 [file mmc1.docx]

**Supplementary Data**

**Supplemental Figure 1:** Scree plot showing eigenvalues for each of the food items, extracted by factor analysis from the data of 48 food items excluding alcoholic beverages and seasonings (A), 42 food items excluding non-alcoholic beverages from A (B), 22 food items finally extracted by factor analysis (C).

A B

C

**Supplemental Figure 2: Participant recruitment and categorization**

Participants enrolled in the Japan Human Milk Study Cohort (n=1,210)

Excluded (n=114)

- Not meeting inclusion criteria (n=5)

- BDHQ for 1–2 months postpartum were not available (n=109)

Eligible participants (n=1,096)

- Missing information

BMI: Pre-pregnancy (n=11), Current (n=2)

Education (n=6)

Household income (n=44)

Maternal smoking habit (n=4)

Delivery experience (n=5)

Mode of delivery (n=4)

Feeding methods (n=2)

Gestational age (n=43)

Maternal health issues: Anemia (n=21), Constipation (n=19),

Rough skin (n=22), Sensitivity to cold (n=61), Mastitis (n=22)
